# Associations and Interactions Between Neuroticism, Adverse Life Events and Health Anxiety: Results From a Large Representative Cohort

**DOI:** 10.32872/cpe.14441

**Published:** 2025-05-28

**Authors:** Thomas Tandrup Lamm, Mimi Yung Mehlsen, Tina Birgitte Wisbech Carstensen, Kaare Bro Wellnitz, Eva Ørnbøl, Thomas Meinertz Dantoft, Per Fink, Marie Weinreich Petersen, Lisbeth Frostholm

**Affiliations:** 1 Research Clinic for Functional Disorders, Aarhus University Hospital, Aarhus, Denmark; 2 Department of Clinical Medicine, Aarhus University, Aarhus, Denmark; 3 Department of Psychology, Aarhus University, Aarhus, Denmark; 4 Center for Clinical Research and Prevention, Bispebjerg and Frederiksberg Hospital, The Capital Region of Denmark, Denmark; Friedrich-Alexander-Universität Erlangen-Nürnberg, Erlangen, Germany

**Keywords:** health anxiety, neuroticism, adversity, Diathesis Stress Model, cohort study, effect moderation

## Abstract

**Purpose:**

Adverse life events and neuroticism have been shown to be associated with health anxiety (HA), but their interaction has not been studied. This study aimed to examine the separate associations as well as the possible interaction effect of neuroticism and adverse life events with HA.

**Method:**

Cross-sectional self-report data originated from a representative Danish population cohort (DanFunD) (*N* = 7,493, 18-72 years, 53% females). Primary measures were HA (Whiteley Index 6 revised), neuroticism (NEO personality Inventory Revised short form), and adverse life events (Cumulative Lifetime Adversity Measure, CLAM). The CLAM was split into illness/death related life events (IDE) and other adverse life events (OAE) to test the specificity of IDEs.

**Results:**

Adjusted ordinal logistic regression models showed positive associations with HA for IDEs (*OR* = 1.05, CI [1.03, 1.08]), OAEs (*OR* = 1.05, CI [1.03, 1.07]) and neuroticism (*OR* = 1.08, CI [1.07, 1.09]). A Wald's test revealed no difference in association with HA for IDEs and OAEs (*p* = .82). Adjusted models showed an interaction effect for neuroticism*IDEs (*OR* = 1.002, CI [1.000, 1.004]), but not for neuroticism*OAEs (*OR* = 0.999, CI [0.996, 1.002]).

**Conclusion:**

Associations with HA were found for both adverse life events and neuroticism. Size of association did not differ for IDEs and OAEs. Only IDEs interacted with neuroticism.

## Highlights

- Neuroticism and adverse life events are associated with health anxiety.
- No difference in effect size between illness/death and other types of adversity was found.
- Illness/death related adversity interact with neuroticism in their association with health anxiety.
- Interaction between Illness/death related adversity and neuroticism was small.

Health anxiety (HA) refers to the experience of recurrent, excessive preoccupations with bodily symptoms and fear of having a serious illness in spite of relatively sparse evidence of illness (Fink et al., 2004). The term covers both mild and transient health-related worries as well as severe and debilitating HA (Longley et al., 2010). Experiences with HA frequently occur in the general population (Sunderland et al., 2013) and if untreated, severe HA has been shown to persist (Fink et al., 2004; olde Hartman et al., 2009) with dire consequences, such as reduced quality of life, more sick leave, more disability pensions, and increased health expenditure (Eilenberg et al., 2015; Fink et al., 2010).

## Models of Severe Health Anxiety

One foundational model to explain why mental disorders occur is the Diathesis-Stress Model (Monroe & Simons, 1991). This model states that mental disorders develop when a pre-disposed individual (individual specific factors) is exposed to an adequate stressor (environmental specific factors). Some studies have tested this model on disorders such as depression and social anxiety, finding significant interactions between adverse life events and diathesis factors such as neuroticism and genetic profiles (Arnau-Soler et al., 2019; Brown & Rosellini, 2011; Howe et al., 2017).

In relation to HA, elements of the Diathesis-Stress Model are reflected in the Cognitive Model of HA, which is the most widely used etiological model of HA (Salkovskis & Warwick, 2001). Here, adverse life events associated with illness and death are thought to contribute to an individual forming inflexible or negative schematic assumption about health and illness. This predisposes individuals to react in catastrophizing ways to future health-related stressors, which can ultimately increase the risk of developing the more severe forms of HA.

According to the Cognitive Model of HA, illness/death related life events (IDEs) play a special role in the development of severe HA and should thus have a stronger association with HA than other adverse life events (OAEs) (e.g. social adversity, natural disasters, violence), which may be associated with increased anxiety and distress but are not specifically linked to health related anxiety.

## Experiences With Illness and Death

The association between adverse life events and HA has been examined in several studies, which indicate higher rates of adverse life events among people that report more HA (Mertz et al., 2023; Reiser et al., 2014; Weck et al., 2014). The specificity of IDEs nevertheless remains unclear (Thorgaard et al., 2018). Some studies indicate a degree of specificity for IDEs (Bailer et al., 2014; Sandin et al., 2004), whereas others show that it is adverse life events, irrespective of type, which are associated with HA (Mertz et al., 2023; Reiser et al., 2014; Weck et al., 2014). Some studies have even indicated a lack of association between HA and IDEs (Barsky et al., 1994; Gehrt et al., 2022).

## Neuroticism

While the Cognitive Model of HA emphasizes the role of stress factors, such as IDEs, less emphasis is placed on the role of predisposing/diathesis factors (Salkovskis & Warwick, 2001). Neuroticism is a trait that reflects an individual’s largely heritable tendency to experience negative emotions (Barlow et al., 2014). It has been argued that neuroticism acts as a central diathesis factor for mood and anxiety disorders (Barlow et al., 2014), and studies have demonstrated that neuroticism can interact with and amplify the effect of adverse life events in relation to depression and stress (Brown & Rosellini, 2011; Howe et al., 2017). Moderate to large associations have furthermore been found between neuroticism and HA (Cox et al., 2000; Taillefer et al., 2003). However, while there are several studies relating severe HA to neuroticism and various types of adverse life events, no studies have examined if these factors interact in their association with HA, as suggested in the Diathesis-Stress Model.

## Aim of the Current Study

To provide insight into the development of HA, the current study aimed to 1) examine the association between HA, neuroticism, and adverse life events related to illness/death (IDEs) as well as other types of adverse life events (OAEs), and 2) to examine if neuroticism and adverse life events interact in the association with an individual’s level of HA.

We hypothesized that: 1) higher levels of neuroticism would be associated with higher levels of HA, 2) higher numbers of adverse life events (both IDEs and OAEs) would be associated with higher levels of HA, 3) IDEs would be more strongly associated with HA than OAEs, 4) there would be an interaction effect between adverse life events (both IDEs and OAEs) and neuroticism in their association with HA, so that scoring higher on adverse life events would cause the association between neuroticism and HA to be amplified.

## Method

### Design and Participants

Data originates from The Danish study of Functional Disorders (DanFunD), which is a representative Danish general population cohort study (Dantoft et al., 2017). 25,368 adults living in the western part of the Greater Copenhagen area of Denmark were randomly drawn from the Danish Civil Registration System based on social security number and invited to participate in the study. They received the invitation via mail to their postal address. 7,493 participants (29.53% of the invited) were included between 2012 and 2015. Participants were excluded if they were 1) pregnant, 2) not born in Denmark, 3) not Danish citizens. Participants answered a large battery of validated questionnaires, covering a variety of biological, psychological, and social domains and underwent a physical health examination. Only measures of relevance to this study will be described (for all measures, see Dantoft et al., 2017).

All participants gave written informed consent. Ethical approval was granted from the Ethical Committee of Copenhagen County (Ethics Committee: 3-2012-0015) as well as the Danish Data Protection Agency (2012–58- 006, 1–16–02-227-16), and the study was conducted in accordance with the Helsinki II Declaration. The current study was not pre-registered.

### Measures

The following measures were used:

#### Dependent Variable

*Health Anxiety* was measured with a revised and abbreviated 6-item version of the Whitely Index (Pilowsky, 1967), called the Whitely Index 6 Revised (WI6-R) (Carstensen, Ørnbøl, Fink, Pedersen, et al., 2020). The WI6-R is a self-report measure, where items are scored on a 0-4 rating scale (from ’Not at all’ to ‘A great deal’), with a range of 0-24. Higher scores indicate more HA. This measure has been validated in the Danish general population, using the DanFunD Cohort (Carstensen, Ørnbøl, Fink, Pedersen, et al., 2020).

#### Independent Variables

*Neuroticism* was measured by the NEO-PI-R short form (sf) (Hansen & Mortensen, 2004). This is an abbreviated version of the Danish 240-item NEO-PI-R. The NEO-PI-Rsf consists of 60 items, which measure the big five traits (neuroticism, conscientiousness, extraversion, agreeableness, openness), though in the current study, only the neuroticism scale was used. In the NEO-PI-Rsf, each trait is measured with 12 items. Answers are given on a 5-point rating scale (‘clearly disagree’ to ‘clearly agree’) with a range of 0-48. Higher scores indicate more neuroticism.

The NEO-PI-Rsf has been validated in a sample of the Danish population showing that the instrument has acceptable psychometric properties (Hansen & Mortensen, 2004).

*Adverse life events* were measured using a Danish version of the Cumulative Life-time Adversity Measure (CLAM) (Seery et al., 2010). This version was translated and validated in a Danish sample using the DanFunD cohort (Carstensen, Ørnbøl, Fink, Jørgensen, et al., 2020). The CLAM is a formative instrument that estimates the occurrence of adverse life events throughout the life span via 37 items, which are divided into 7 categories: 1) Own-illness or injury, 2) Loved one’s illness or injury, 3) Violence, 4) Bereavement, 5) Social/environmental stress, 6) Relationship stress, 7) Disaster. For each event, three instances of the event and one period can be recorded (from time point A to B). One self-described event can also be recorded. For each item, a score is calculated by adding all recorded instances into a single score (max 4 events per item). Higher scores indicate more adverse life events.

A total score for each category of adverse life events can be formed by combining the scores of items for the specific event category. A total lifetime adversity score can also be constructed by summing the total scores from all event categories. For the current study, an “illness/death related life events” (IDEs) variable was computed by combining the categories: “own-illness or injury”, “loved one’s illness or injury” and “bereavement” into a single score (13 items, range 0-52). To assess the effect of the remaining adverse life events, an “other adverse life events” (OAEs) variable was computed by combining the remaining CLAM categories, including “violence”, “social/environmental stress”, “relationship stress”, “disaster” (26 items, range 0-104).

#### Covariates

Covariates were chosen based on Directed Acyclic Graphs (Moffa et al., 2017). This is further elaborated upon in the following section ‘Analysis’. The following covariate measures were used:

*Self-reported social status* was measured with a validated item asking individuals how they would rate their own social position in society on a ranking scale from 1-10, which was scored on an ordinal scale from 1 to 10, with 10 representing the highest possible self-reported social status (Demakakos et al., 2008).

*Physical fitness* was tested using The Danish Step Test (Aadahl et al., 2013), which is a validated measure of cardiorespiratory fitness. The test is conducted by asking participants to step up and down on a step bench for a maximum of 6 minutes following instructions from a computer. Maximal oxygen consumption (VO_2_max) is estimated based on how long participants can keep up with the pace of instructions and is then calculated based on known principles of the energy costs. Physical fitness was scored from 0-20, where a higher score indicated better physical fitness (Aadahl et al., 2013; Dantoft et al., 2017).

*Sleep problems* were measured using 2 items (“How often do you have problems falling asleep?” and “How often do you wake up too early compared to how long you would like to sleep?”), which have been used in a previous population cohort (Byberg et al., 2012; Dantoft et al., 2017). Both items were scored from 1-4 using the following categories: 1 = “once per month or rarer”; 2 = “two to four times per month”; 3 = “once or more times a week”; 4 = “daily”. In the current study, sleep problems were binarily rated as having “sleep problems” if participants scored above 2 on either of the items, and “no sleep problems” if they did not.

### Analysis

Descriptive analyses consisted of counts (%), range, mean (*SD*), or median (IQR) depending on the variables and their empirical degree of skew. Because of long tails on the distribution of IDEs and OAEs, the tails were shortened by reducing all values above a given threshold to the threshold value (IDEs threshold = 10; OAEs threshold = 7).

All hypotheses were analyzed using both crude and adjusted ordinal logistic regression models with HA as an ordered outcome. Hypotheses were tested based on the adjusted estimates as they provide minimally confounded estimates, but crude estimates were also reported to facilitate comparison with other studies.

Adjustment variables were chosen based on Directed Acyclic Graphs (DAGs) (Moffa et al., 2017), and the minimal set of adjustment variables was found using the free online software Daggity (Textor et al., 2016). DAGs were drawn by a subset of the author group (TTL, TC, KBW, MWP, LF) and included neuroticism, IDEs, OAEs, age (in years), physical activity, physical symptoms, sex (male/female), sleep problems, and socio-economic status. The minimal set of adjustment variables consisted of sleep problems, physical activity, and self-reported social status as well as neuroticism, IDEs, and/or OAEs depending on primary predictor. Thus, Hypotheses 1, 2, and 3 were all evaluated based on parameters from the same adjusted ordinal logistic regression analysis. Syntax for the DAGs can be found in Appendix A.

Odds ratio (*OR*) was used as a measure of association, and hypotheses were evaluated at alpha level .05 by interpreting 95% confidence intervals (CI) and whether or not CI overlapped with 1.

Hypotheses 1 and 2 that HA was associated with neuroticism, IDEs, and OAEs were evaluated based on the adjusted CI for the associations of neuroticism, IDEs, or OAEs with HA. *OR* > 1 indicated that higher neuroticism or more IDEs/OAEs were associated with greater odds of HA. Hypothesis 3 that IDEs were more strongly associated with HA than OAEs was evaluated by direction and degree of overlap of the adjusted CIs of IDEs and OAEs and was formally tested using Wald's test. Hypothesis 4 that neuroticism and IDEs or OAEs interacted in the association with HA was tested by adding either neuroticism*IDEs or neuroticism*OAEs to the adjusted model used for Hypotheses 1, 2, and 3.

It was furthermore examined to which degree IDEs had a specific association with HA, or if similar associations could be found in relation to more general measures of anxiety or depression. To do this, supplementary analyses were conducted using the SCL-90 depression and anxiety scales as the dependent variables (description of measures in Appendix B), and IDEs, neuroticism and their interaction as the independent variables. Both crude and adjusted models were estimated, using the same set of covariates as the ordinal logistic regression model predicting HA.

All independent variables and covariates were median centered. Proportional odds assumptions were tested using Brant tests (Brant, 1990). Linearity of continuous covariates was checked by expanding the model with natural cubic splines with five knots at the 5th, 27.5th, 50th, 72.5th, and 95th percentiles, following the recommendations by (Harrell, 2015). All analyses were run in STATA version 18.0 (StataCorp, 2023).

## Results

The studied population consisted of *N* = 7,493 participants aged 18-72, of which 53% were females. Rates of missing data were below 1.7% for all measures with a completion rate of 99.4% for HA, 98.2% for neuroticism, and 98,8% for adverse life events. Further descriptive statistics for the analyzed cohort can be found in Table 1.

**Table 1:** Sample Characteristics

Variable	*N* (%)	*M* (*SD*)^a^/ Median (IQR)^b^	Range	*N* missing (%)
Sex				
Males	3456 (46)			
Females	4037 (54)			
Age^b^	7493	54 (44-63)	18-72	
Self-rated social status^a^	7407	6.62 (1.40)	1-10	86 (1.15)
Sleep problems				60 (0.80)
No	6371 (86)			
Yes	1062 (14)			
Physical fitness^a^	6419	9.52 (2.73)	1-19	1074 (14.33)
Health anxiety^b^	7454	2 (0-5)	0-24	39 (0.52)
Neuroticism^a^	7365	16.47 (7.48)	0-46	128 (1.71)
Adverse life events, total^b^	7405	5 (3-8)	0-35	88 (1.17)
Illness/death related events^b^	7361	4 (2-6)	0-19	132 (1.76)
Other adverse life events^b^	7405	1 (0-2)	0-25	88 (1.17)

*Note*. Mean (standard deviation) or Median (interquartile range) was reported selectively based on the empirical distribution of each variable. For variables that were normally distributed, mean (*SD*) was reported. For variables that were non-normally distributed, median (IQR) was reported. Reporting format is denoted in the superscript.

^a^Mean, *SD*. ^b^Median, IQR.

Testing Hypotheses 1 and 2, which stated that there would be a positive association between HA and OAEs, IDEs, and neuroticism, revealed a positive association with HA for neuroticism, IDEs, and OAEs. This indicated that having a higher score on these variables was associated with higher odds of reporting more HA (see Table 2). The size of these effects varied. To enhance interpretability, the standard deviation (*SD*) and interquartile range (IQR) were used to estimate the difference in odds of scoring higher on HA between two individuals where one scored one *SD* or quartile above the other on the dependent variables.

**Table 2:** Ordinal Logistic Regression Models and Interaction Effects for the Association Between Neuroticism, Adverse Life Events, and Health Anxiety

Independent variable	*OR* [95% CI]	*Z*	*p*	*N*
Neuroticism, cru	1.09 [1.09, 1.10]	31.22	< 0.01	7362
Neuroticism, adj^a^	1.08 [1.07, 1.09]	24.24	< 0.01	6169
Illness/death related life events, cru	1.05 [1.03, 1.07]	6.59	< 0.01	7348
Illness/death related life events, adj^b^	1.05 [1.03, 1.07]	5.74	< 0.01	6169
Other adverse life events, cru	1.12 [1.10, 1.15]	11.02	< 0.01	7391
Other adverse life events, adj^c^	1.05 [1.03, 1.08]	4.48	< 0.01	6169
Illness/death*neuroticism, cru	1.002 [1.000, 1.005]	2.60	< 0.01	7261
Illness/death*neuroticism, adj^d^	1.002 [1.000, 1.004]	1.96	0.05	6169
Other adverse*neuroticism, cru	1.000 [0.997, 1.002]	0.08	0.93	7303
Other adverse*neuroticism, adj^e,f^	0.999 [0.996, 1.002]	-0.25	0.82	6169

*Note.* Cru = crude; Adj = adjusted; *OR* = odds ratio. *OR* values should be interpreted as the cumulative odds of scoring higher on the WI6-R for each additional point on neuroticism (range: 0-48), Other adverse life events (range: 0-104), Illness/death related life events (range: 0-52). BRANT tests for proportional odds were non-significant in all cases except for the crude model for the interaction between neuroticism and illness/death related life events, where a BRANT test showed a *p* = .023 for the overall model. Nevertheless, variable level tests were all *p* > .05. Consequently, we chose to regard the assumption of proportional odds as sufficiently fulfilled.

^a^illness/death related life events, other adverse life events, physical activity, sleep problems, self-reported social status. ^b^neuroticism, other adverse life events, physical activity, sleep problems, self-reported social status. ^c^illness/death related life events, neuroticism, physical activity, sleep problems, self-reported social status. ^d^other adverse life events, physical activity, sleep problems, self-reported social status. ^e^illness/death related life events, physical activity, sleep problems, self-reported social status. ^f^Interaction effects in the last four rows are based on ordinal logistic regression models with interaction terms included. To see the estimated parameters of all variables included in these models, see Appendix C.

For neuroticism, it was estimated based on the adjusted model that an individual scoring one *SD* higher on neuroticism would have 74% higher odds of scoring higher on HA. For IDEs, it was estimated based on the adjusted model that an individual scoring one quartile higher would have a 10% higher odds of scoring higher on HA. For OAEs, this was 5%.

Hypothesis 3 was examined by testing to which degree there was a difference in the strength of the association with HA for the IDEs and OAEs variables. A Wald's test (χ^2^(1) = 0.05, *p* = .82) indicated that there was no difference in the effect sizes between IDEs and OAEs and that both variables had similar *OR*s and almost identical CIs (see Table 2).

Testing Hypothesis 4 regarding the interactions between neuroticism and the two types of adverse life events (IDEs and OAEs), a small interaction effect between neuroticism and IDEs in their association with HA was found. No interaction was found for neuroticism and OAEs (see Table 2). This indicates that IDEs amplified the association between neuroticism and HA, rather than the effect of IDEs and neuroticism simply being additive. A more tangible effect size was calculated by using the model parameters to estimate the difference in odds of scoring higher on HA for two individuals where one individual scored one *SD* higher on neuroticism and one quartile higher on IDEs. The higher scoring individual was estimated to have a 2% higher odds of scoring higher on HA.

To further ascertain the specificity of the interaction between IDEs and neuroticism for HA, similar ordinal logistic regression models were run with depression and anxiety measures as the outcome instead of HA (see Appendix D). These models indicated that both neuroticism and IDEs were associated with anxiety and depression scores. The effect sizes of the associations were slightly larger for neuroticism than what was found in the model predicting HA, and slightly smaller for IDEs. In contrast to models predicting HA, no interaction effect was found when predicting anxiety or depression from the interaction between neuroticism and IDEs (*p* > .05).

## Discussion

### Summary of Findings

This population-based study found positive associations between health anxiety and illness/death related life events, other adverse life events, and neuroticism (Hypotheses 1 and 2). It further indicated that illness/death related life events did not have a stronger association with HA than other types of adverse life events (Hypothesis 3). Finally, a small interaction effect was shown for illness/death related life events, which amplified the effect of neuroticism on health anxiety. No such interaction with neuroticism was found for other adverse life events (Hypothesis 4).

### Associations Between Health Anxiety, Adverse Life Events, and Neuroticism

The finding that both neuroticism and adverse life events were positively associated with HA is in line with other studies on the association between HA and different types of adverse life events (Mertz et al., 2023; Reiser et al., 2014; Thorgaard et al., 2018; Weck et al., 2014) and neuroticism (Cox et al., 2000; Taillefer et al., 2003). It should also be noted that the positive associations between neuroticism and adverse life events have been found for other types of mental disorders in previous studies and is thus not likely to be specific to HA (Hogg et al., 2023; Kotov et al., 2010). Similarly, in the current study, IDEs had associations to depression and anxiety which were comparable in size to what was found in relation to HA.

It should also be considered that the estimated effect of neuroticism appeared to be larger than the effects of both OAEs and IDEs. This difference in effect may indicate that dispositional factors could be more strongly associated with the development of HA than adverse life events.

### The Specificity of IDEs

The lack of specificity for IDEs found when testing Hypothesis 3 is in line with the few other studies that have not been able to identify difference in the size of the association with HA for OAEs and IDEs (Barsky et al., 1994; Mertz et al., 2023; Reiser et al., 2014; Weck et al., 2014). This is also reflected in the review by Thorgaard et al. (2018) which showed that several studies demonstrate associations with HA for both event types. The inconsistency of the association between IDEs and HA could be interpreted as incongruent with the Cognitive Model of HA, which proposes that IDEs should play a specific causal role in the development of severe HA.

### Interactions Between Adverse Life Events and Neuroticism

The current study is the first to test for an interaction effect between neuroticism and adverse life events in relation to HA, finding an interaction between neuroticism and IDEs, which does not appear for the association between neuroticism and OAEs. This appears to be consistent with other studies examining diathesis-stress interactions for other mood and anxiety disorders that have found similar significant interaction effects (Arnau-Soler et al., 2019; Brown & Rosellini, 2011; Howe et al., 2017) and aligns with what would be expected based on the Diathesis-Stress Model. Furthermore, supplementary analyses showed that an interaction between IDEs and neuroticism could not be identified when examining the association with more general depression and anxiety. Thus, the interaction effect between neuroticism and IDEs seemed to only occur in relation to HA.

Importantly, the size of the interaction effect was small. Scoring highly on both neuroticism and IDEs resulted in only 2% higher odds of scoring higher on HA. The size of this association puts the theoretical and clinical implications of this finding into question.

### Implication and Future Work

The results of this study indicate that both diathesis and stress factors are associated with HA, consistent with the Diathesis-Stress Model (Monroe & Simons, 1991), though inconsistent evidence was found for the specificity of IDEs in relation to HA, which is implied in the Cognitive model of HA (Salkovskis & Warwick, 2001).

To more robustly establish the causal role of OAEs, IDEs and neuroticism in relation to HA, future studies should preferably use prospective designs. Current findings do not support the importance of future studies examining IDEs as a separate category instead of measures of cumulative adversity (Seery et al., 2010).

Furthermore, the current model supports the importance of dispositional factors in relation to HA, as neuroticism was shown to have the strongest association with HA. Future theoretical work could focus on integrating these factors in the cognitive model of HA (Salkovskis & Warwick, 2001).

The knowledge about the relative importance of neuroticism and adverse life events could inform clinical work with HA patients and may be integrated in case formulation, psychoeducation or during psychotherapy (Bagby et al., 2016; Buwalda & Bouman, 2008).

### Strengths and Limitations

The strength of the current study is its use of validated self-report measures in a large representative sample of the general population. All analyses are theoretically driven, testing popular models within the field, and the current study is also the first to test the interactions between neuroticism and adverse life events in relation to HA.

The study is limited by its cross-sectional design, which inhibits causal inference (Kraemer et al., 1997). While it is well established that both adverse life events and neuroticism are prospective risk factors for various types of mental disorders (Jeronimus et al., 2016; Li et al., 2016), bi-directional effects have also been shown, where mental disorder can increase both rates of stressful life events (Rnic et al., 2023) and levels of neuroticism (Ormel et al., 2013). The retrospective nature of the CLAM also makes it vulnerable to recall bias, whereby current state effects may modify the reported frequency or significance of past events (Lalande & Bonanno, 2011).

Another limitation is related to the use of a self-reported measure for HA, as distress reported via self-report may not always correspond to actual clinically significant distress (Hedman et al., 2015). Thus, the current findings could be supplemented in future studies using dichotomous classifications of HA set via clinical interviews to better estimate the clinical implication of findings.

Finally, the aggregated adverse life event variables used in the current study (IDEs and OAEs) were created by combining specific subscales from the CLAM for this specific study (Carstensen, Ørnbøl, Fink, Jørgensen, et al., 2020). As such, these subdivisions have not been formally validated. However, as the CLAM is a formative measure, this should not pose a threat to validity.

## Conclusion

Using data from the large representative DanFunD cohort, the current study has found positive associations between health anxiety and neuroticism, illness/death related life events and other adverse life events. The findings show that illness/death related life events did not have a stronger relation to health anxiety than other adverse life events. While a specific interaction effect was found between illness/death related life events and neuroticism in relation to health anxiety, the size of this interaction was small, and the clinical significance of this finding remains unclear.

## Supplementary Materials

The Supplementary Materials contain the following items (for access, see Lamm et al., 2025S):

- Appendix A: Syntax of Directed Acyclic Graphs (DAGs)
- Appendix B: Ordinal logistical regression models of the association between neuroticism, adverse life events, and health anxiety with interactions
- Appendix C: Description of measures used in supplementary analyses and descriptive statistics
- Appendix D: Results of supplementary analyses

LammT. T.
MehlsenM. Y.
CarstensenT. B. W.
WellnitzK. B.
ØrnbølE.
DantoftT. M.
FinkP.
PetersenM. W.
FrostholmL.
 (2025S). Supplementary materials to "Associations and interactions between neuroticism, adverse life events and health anxiety: Results from a large representative cohort"
[Online appendices]. PsychOpen. 10.23668/psycharchives.16215
PMC1215222540510544

## Data Availability

All data collected in the current study are confidential, and data can therefore not be made available. The code used for statistical analysis and other study materials can be shared upon reasonable request.

## Reporting Guidelines

The current manuscript was formatted according to the JARS-Quant reporting guidelines for Quantitative Research Designs.

## Preregistration

The current study was not pre-registered. The current study is not a replication study.
